# The Attempted Assassination

**Published:** 1881-08

**Authors:** 


The recent attempt on the life of the President was an outrageous occurrence, and is a terrible commentary on the social and moral status of the present day. The various exhibitions of this degeneracy that have heretofore taken place have been marked by disturbances which ended more or less in anarchy and general confusion. Circumstances, and probably the peculiar disposition of the American people, have averted the more extreme evils that might attend such an event, while the threatened danger has united the nation in an effort to quiet confusion and allay apprehension.

While we are called upon to observe the governmental and social obliquity, we are in the same connection brought face to face with a professional evil. Nothing could be more disgraceful than the doctor's quarrel, which occurred not long since, over the wounded victim.

What could tend more to bring disrepute to the profession than such conduct on such a momentous and national crisis? It would appear that little reliance could be reasonably placed in doctors on great emergencies, and, therefore, none on smaller ones. We will not attempt to discuss the causes that led to the medical comedy of the White House—all who are acquainted with the parties concerned are prepared to entertain some idea as to the operating influences.

The time should come for the peace and harmony of the medical profession when some broad and liberal basis of unanimity and fraternal fellowship may be established that can resist the combined assaults of avarice, demagogueism and humbuggery. Until this time comes the disgraceful scenes of competition will be often rehearsed to the detriment of the profession in general.

